# Novel Synonymous and Deep Intronic Variants Causing Primary and Secondary Pyruvate Dehydrogenase Complex Deficiency

**DOI:** 10.1155/2024/1611838

**Published:** 2024-03-25

**Authors:** Helene Bruhn, Karin Naess, Sofia Ygberg, Lucía Peña-Pérez, Nicole Lesko, Rolf Wibom, Christoph Freyer, Henrik Stranneheim, Anna Wedell, Anna Wredenberg

**Affiliations:** ^1^Department of Medical Biochemistry and Biophysics, Karolinska Institutet, 17177 Stockholm, Sweden; ^2^Centre for Inherited Metabolic Diseases, Karolinska University Hospital, 17176 Stockholm, Sweden; ^3^Department of Child Neurology, Karolinska University Hospital, 17176 Stockholm, Sweden; ^4^Department of Molecular Medicine and Surgery, Karolinska Institutet, 17177 Stockholm, Sweden; ^5^Science for Life Laboratory, Karolinska Institutet, 17177 Stockholm, Sweden

## Abstract

Pyruvate dehydrogenase complex deficiency (PDCD) is a defect of aerobic carbohydrate metabolism that causes neurological disorders with varying degrees of severity. We report the clinical, biochemical, and molecular findings in patients with primary and secondary PDCD caused by novel atypical genetic variants. Whole-genome sequencing (WGS) identified the synonymous variants c.447A>G, p.(Lys149=) and c.570C>T, p.(Cys190=) in pyruvate dehydrogenase E1 subunit alpha 1 (*PDHA1*), the deep intronic variants c.1023+2267G>A and c.1023+2302A>G in pyruvate dehydrogenase complex component X (*PDHX*), and c.185+15054G>A in thiamine pyrophosphokinase (*TPK1*). Analysis by Sanger and RNA sequencing of cDNA from patient blood and/or cultured fibroblasts showed that the synonymous variants in *PDHA1* lead to aberrant splicing and skipping of exons 5 and 5-6 in one of the patients and transcripts lacking exon 6 in the other. The deep intronic variants in *PDHX* and *TPK1* lead to insertion of intronic sequence in the corresponding transcripts. The splice defects in *PDHA1* were more pronounced in cultured fibroblasts than in blood. Our findings expand the spectrum of pathogenic variants causing PDCD and highlight the importance of atypical variants leading to aberrant splicing. The severity of the splice defects and resulting biochemical dysfunction varied between tissues, stressing the importance of performing biochemical and transcript analysis in affected tissues. The two males with hemizygous synonymous *PDHA1* variants have a mild phenotype and higher PDH enzyme activity than expected, which is consistent with aberrant but leaky splicing with a proportion of the transcripts remaining correctly spliced.

## 1. Introduction

Pyruvate dehydrogenase complex deficiency (PDCD) is a disorder of mitochondrial carbohydrate oxidation, resulting in abnormal energy metabolism of the cell. It is often included in the group of mitochondrial disorders, although there is no defect of the oxidative phosphorylation system. The phenotypic spectrum is broad, ranging from lethal lactic acidosis neonatally to a chronic neurological condition with structural abnormalities in the central nervous system (CNS) without systemic acidosis. Symptoms may include signs of neurological dysfunction such as developmental delay, intermittent ataxia, hypotonia, abnormal eye movements, and seizures. The most common form of primary PDCD is caused by variants in the gene encoding pyruvate dehydrogenase E1 subunit alpha 1 (*PDHA1*) on the X chromosome. Although it is an X-linked disorder, many females, depending on variant type and the pattern of X chromosome inactivation [1], present with severe symptoms. Other rarer forms are caused by variants in the genes encoding other components of the complex, pyruvate dehydrogenase E1 subunit beta (*PDHB*), E2 component of pyruvate dehydrogenase complex (*DLAT*), E3 component of pyruvate dehydrogenase complex (*DLD*), and pyruvate dehydrogenase complex component X (*PDHX*), which are all inherited in an autosomal recessive manner. In addition, variants in more than 20 genes cause secondary PDCD, due to assembly defects or metabolism of cofactors such as thiamine or lipoic acid. One example is thiamine pyrophosphokinase (TPK) deficiency caused by variants in the gene *TPK1* [2]. TPK catalyzes the conversion of thiamine (vitamin B1) to thiamine pyrophosphate (TPP). TPP is a cofactor for several enzymes, for example, transketolase, pyruvate dehydrogenase, and *α*-ketoglutarate dehydrogenase. Secondary PDCD may have completely different treatment options despite a similar symptomatology, which makes timely identification of the disease-causing gene important.

Next-generation sequencing has greatly improved the detection of variants, but the biological and clinical interpretation of many variants still remains challenging. Particularly difficult are novel variants in intronic regions as well as synonymous variants, which may affect splicing and thus are significant contributors to human disease. In fact, single nucleotide variants (SNVs) at canonical splice junctions constitute about 10% of reported disease-causing variants and may lead to retention of intronic DNA in mRNA or to entire exons being spliced out [3]. However, the recognition of the 5′ (donor) and 3′ (acceptor) splice sites by the spliceosome is not enough for correct splicing. Additional regulatory factors such as exonic and intronic splice enhancers (ESEs and ISEs, respectively) and silencers (ESSs and ISSs) are needed, and they are particularly important in genes with weak canonical splice sites. About 1,6% of pathogenic missense variants are actually estimated to lead to aberrant splicing [3]. Exonic variants may inactivate an ESE or create an ESS element in the sequence, leading to insufficient exon inclusion or altering the secondary structure of mRNA and thereby affecting its stability [4–7]. In fact, almost 600 synonymous variants have been reported as pathogenic or likely pathogenic in 385 genes in ClinVar [8] (accession date August 17, 2023). The term synonymous is thereby in some sense misleading, since the variant may have an effect on DNA, RNA, and/or protein level even if it looks silent at the codon level. Recently, unsense variant was introduced in Variation Ontology (VariO), where the definition is a substitution in the mRNA coding region that affects gene expression and protein production without introducing a stop codon in the variation site [9]. Deep intronic variants can lead to pseudoexon inclusion by activating a noncanonical splice site, i.e., a variant which creates a novel donor site that activates an existing acceptor splice site. Pseudoexon inclusion may also be caused by a variant which changes a splicing enhancer or a silencing element in the intron. Thus, deep intronic variants have been reported to cause disease in over 75 different genes [10].

We report here the clinical, biochemical, and molecular findings in patients with primary and secondary PDCD caused by novel synonymous and deep intronic variants in *PDHA1*, *PDHX*, and *TPK1*. We demonstrate by complementary DNA (cDNA) analysis and/or RNA sequencing (RNAseq) that the identified variants cause exon skipping or intronic sequence inclusion in the mRNA of the affected genes. We hereby expand the diversity of genetic variants causing PDCD and emphasize the importance of atypical variants leading to defective splicing.

## 2. Materials and Methods

### 2.1. Editorial Policies and Ethical Considerations

The study has been approved by the regional ethical board in Stockholm, Sweden (2008/351-31, 2021-02050). Informed consent was obtained from all the patients or their families.

### 2.2. Patients

The patients were referred to the Centre for Inherited Metabolic Diseases at Karolinska University Hospital, for a muscle biopsy and mitochondrial investigations, during the years 1991-2017. Physical examination and compilation of medical history was performed as part of the clinical workup.

### 2.3. Investigations of Muscle Tissues and Cultured Fibroblasts

All patients underwent a percutaneous muscle biopsy from *m. tibialis anterior* using a conchotome as described previously [11]. Also, a skin biopsy was taken in connection to the muscle biopsy. Patient 5 (P5) was investigated on two occasions, 1991 and 1994, to determine mitochondrial ATP production rate (MAPR), mitochondrial respiratory chain (MRC) enzyme activities, and citrate synthase activity, as described previously [12, 13]. The remaining patients were investigated 2003-2017 with an improved set of the same methods [11]. Cultures of primary skin fibroblasts from skin biopsies were established and cultured at 37°C/5% CO_2_ atmosphere in standard high-glucose DMEM-GlutaMAX media (Gibco Life Technologies), supplemented with 10% FBS (Gibco Life Technologies). Cultured fibroblasts from patient 1 (P1) and patient 2 (P2) were sent to the Oxford Medical Genetics Laboratories for analysis of pyruvate dehydrogenase activity. Total PDC activity was measured after maximal activation with dichloroacetate, using [1-^14^C]-pyruvate as substrate, as described previously [14].

### 2.4. DNA Analyses

Genomic DNA was isolated from blood (P1 and P4), fibroblast cultures (P3 and P5), or skeletal muscle (P2), using the QIAamp DNA Midi or Mini Kits, (Qiagen), respectively. Genomic DNA from parents was isolated from blood using the QIAamp DNA Midi Kit (Qiagen). Whole-genome sequencing (WGS) of genomic DNA from patients P1, P2, P3, and P5 and the parents of P5 (only case run as WGS trio) was performed to a sequencing depth of 30× mean coverage using a HiSeq X (Illumina) or NovaSeq 6000 sequencing instrument (Illumina) after library preparation with Illumina TruSeq DNA PCR-free Kit for DNA from blood and fibroblasts and NxSeq AmpFREE Low DNA Library Kit for DNA from muscle. This was followed by in-house bioinformatic analysis, using the mutation identification pipeline (MIP) as earlier described [15, 16]. All known nuclear genes associated with mitochondrial disease or other inherited metabolic diseases were analyzed, using precompiled gene panels that are available at https://www.karolinska.se/for-vardgivare/karolinska-universitetslaboratoriet/centrum-for-medfodda-metabola-sjukdomar/genetisk-diagnostik/. For P2 where DNA from muscle was used, sequence data from mitochondrial DNA (mtDNA) was also analyzed. Exons or introns containing the variants in *PDHA1*, *PDHX*, and *TPK1* identified by WGS were amplified and sequenced by Sanger sequencing in samples from genomic DNA from the patient and parents, using M13-tailed primers, under standard conditions. All amplicons were sequenced with BigDye Terminator cycle sequencing chemistries (Applied Biosystems) on a 3500xl DNA sequencer (Applied Biosystems). The Sanger sequence data was compared to NM_022445.3 for *TPK1*, NM_003477.3 for *PDHX*, and NM_000284.4 for *PDHA1*, the MANE Select transcripts [17] for the genes.

### 2.5. Population Frequency and *In Silico* Analyses of Detected Variants

Variant frequencies were obtained from gnomAD (https://gnomad.broadinstitute.org/, v4.0.0) and a local database containing WGS data from a total of 11533 analyzed human samples. SpliceAI [18] and Alamut Visual Plus version 1.2 (© 2021 SOPHiA GENETICS) were used for *in silico* prediction of the impact of splicing of the variants. Alamut has a splicing module that integrates several splice prediction programs: SpliceSiteFinder-like (SSF) [19], MaxEntScan (MES) [20], NNSPLICE (BDGP: Splice Site Prediction by Neural Network (http://fruitfly.org)), and GeneSplicer [21] for prediction of changes at donor and acceptor sites. It also includes the modules exonic splicing enhancer finder (ESEFinder) [22, 23], which present binding scores for the motifs of the four different human serine/arginine-residue proteins (SR proteins) SRSF1 (SF2/ASF), SRSF2 (SC35), SRSF5 (SRp40), and SRSF6 (SRp55); RESCUE-ESE [24] for ESE binding site detection; and EX-SKIP [25] that compares the ESE/ESS profile for the wild-type and mutated sequence to predict possible exon skipping. For prediction of the impact of the missense variant, Polyphen [26], SIFT [27], and Combined Annotation Dependent Depletion (CADD) [28] were used.

### 2.6. RNA Analyses

RNA was isolated from cultured fibroblasts using RNeasy mini kit (Qiagen) and from whole blood using PAXgene RNA Blood Kit (Qiagen). In some RNA experiments, emetine was added to the fibroblast culture medium at a concentration of 100 *μ*g/mL seven hours before harvesting, to inhibit nonsense-mediated mRNA decay [29].

cDNA was synthesized from RNA isolated from fibroblasts and blood using High Capacity cDNA Reverse Transcription kit (Applied Biosystems). For validation of the variants c.447A>G and c.570C>T in *PDHA1* (in exons 5 and 6, respectively), a forward primer in exon 1, PDHA1_cDNA_1F (5′-CTCCTGGGTTGTGAGGAGTC), and a reverse primer in exon 7, PDHA1_cDNA_7R (5′-CTGCCATGTTGTAAGCTTCG), both M13-tailed, were designed for amplification of a 680 bp + 36 bp (M13) wild-type cDNA product. For validation of the variants c.1023+2267G>A and c.1023+2302A>G in *PDHX* (both in intron 9), a forward primer in exon 6, PDHX_cDNA_6F (5′-CAACTCCTGGACAACCCAAT), and a reverse primer in exon 11, PDHX_cDNA_11R (5′-TGCAGTAAATTCGTCGATGC), both M13-tailed, were designed for amplification of a 500 bp + 36 bp (M13) wild-type cDNA product. For validation of variant c.185+15054G>A (positioned in intron 5) in *TPK1*, allele-specific primers in exon 4, TPK1_cDNA_4Fwt (5′-GGTGCCAACCGCTTATATG**A**) and TPK1_cDNA_4Fmut (5′-GGTGCCAACCGCTTATATG**G)**, M13-tailed, were designed, where the last nucleotide in the primers (bold A or G) correspond to position c.161. The patient harbors the variant c.161A>G in a heterozygous state in trans with c.185+15054G>A. These primers were paired in different amplification reactions with a reverse primer in exon 7, TPK1_cDNA_7R (5′-CCCAGTGTCACGATCACATC) to amplify the allele of the father and the mother separately. All cDNA PCR reactions were run under standard conditions except for the allele-specific amplification of *TPK1*, which was run with an annealing temperature of 67°C. PCR products of cDNA were analyzed by electrophoresis on a 1-2% agarose gel. For PCR reactions resulting in the amplification of more than one fragment, the separate fragments were purified on QIAQuick gel purification columns (Qiagen) before sequencing. All amplicons were sequenced with BigDye Terminator cycle sequencing chemistries (Applied Biosystems) on a 3500xl DNA sequencer (Applied Biosystems).

RNA sequencing libraries were generated with Illumina Stranded mRNA library preparation kit (Illumina) for samples from P2, P3, P4, and P5 and with Illumina TruSeq Stranded mRNA (Illumina) for samples from P1 according to the instructions from the manufacturer. Briefly, 350 ng (P2 blood), 300 ng (P2 fibroblasts, P3, P4, and P5), or 500 ng (P1) mRNA was purified from total RNA by poly-A capture, using magnetic beads with poly-T oligos. This was followed by fragmentation of the mRNA and conversion into cDNA through first- and second-strand synthesis. A-tailing was performed and followed by an adaptor ligation including an index sequence and finally amplification by 11 (P2 fibroblasts, P3, P4, and P5) or 15 cycles (P2 blood and P1) of PCR. The finished libraries were validated by measuring concentration and average library length using Quant-iT dsDNA HS (Invitrogen) and TapeStation HS D1000 assay (Agilent) (P2 fibroblasts, P3, P4, and P5), Qubit dsDNA HS (Invitrogen) and TapeStation HS D1000 assay (Agilent) (P1), and BioAnalyzer High Sensitivity DNA kit (Agilent) (P2 blood). Sequencing was done on NovaSeq 6000 (Illumina) using paired-end 150 readout. Demultiplexing was done using bcl2fastq Conversion Software v2.20 (Illumina) (P2 fibroblasts, P3, P4, and P5) and bcl2fastq Conversion Software v2.20.0.422 (Illumina) (P2 blood and P1).

Processing of RNAseq data was performed using MIP-RNA (https://github.com/Clinical-Genomics/MIP), box plots were plotted with R package ggplot2 [30], and sashimi plots with ggsashimi [31].

## 3. Results

### 3.1. Clinical Information

P1 (male) had multiple food intolerances and allergies from an early age. He also had frequent infections. From 11 months of age, recurrent periods of tiredness, strabismus, nystagmus, and ptosis appeared. These periods later also included unsteady gait/balance problems, and they were often concomitant to infections. Between these periods, he developed normally. Investigations regarding myasthenic syndromes were negative. EEG was normal. MRI of the brain was normal at the age of 15 months. Lactate was slightly increased in blood (3,7 mmol/L, ref.<2, 3) and in cerebrospinal fluid (CSF) (3,2 mmol/L, ref.<2, 3). After PDCD diagnosis, a ketogenic diet was tried for one year with some improvement regarding the periods of deterioration. Due to side effects of the diet (episodes of sweating and visual hallucinations), the treatment was terminated. At the current age of seven years, the patient has a mild motor delay and gets easily exhausted. He has no cognitive impairment. In the last year, he has experienced attacks of hyperacusis and has had periods of ptosis, unilateral facial weakness, and unsteady gait. He is treated with thiamine and has no dietary restrictions.

P2 (male) was reported to be healthy until seven years of age when he came to medical attention due to right-sided weakness and slurred speech. Computer tomography w/wo angio were normal, but CSF analysis indicated mildly elevated lactate (3,2 mmol/L). MRI showed cerebellar hyperintensities. Whole exome sequencing (WES), mtDNA sequencing, and mtDNA Southern blot in DNA from muscle did not identify a genetic cause. Follow-up MRI showed a complete regression of the cerebellar hyperintensities. At the age of nine years, he was admitted to intensive care due to a respiratory failure and a hypertonic crisis. The MRI of the brain now mainly had altered signal in the brainstem. Again, CSF and plasma lactate were increased. The boy recovered, and the follow-up MRI was normal. During the following year, the boy had repeated episodes of high blood pressure, choreatic movements, altered mental status including severe anxiety, and increased plasma lactate. He was then put on thiamine, biotin, Q10, riboflavin, and vitamin E, which led to a significant clinical improvement. After PDCD diagnosis, a ketogenic diet was added, followed by further stabilization.

P3 (female) is currently 37 years old. She had a global developmental delay from an early age and has severe learning disabilities. Throughout life, she has had muscular hypotonia and muscle weakness. From 14 years of age, she had episodes of hyperventilation, associated with tiredness and anxiety, sometimes also with short epileptic seizures. The episodes were often concomitant to infections. Lactic acidosis was seen during these episodes, with blood lactate 8-10 mM (ref.<2, 3 mM). Mitochondrial disease was suspected, and a muscle biopsy was performed at 17 years of age.

P4 (female) is the older sister of patient 3. She has had a similar history of disease as her younger sister. At her current age of 42 years, she has severe learning disabilities, hypotonia, and mobility impairment.

P5 (female) initially had normal psychomotor development, but from six months of age, developmental delay was observed. She ended up with severe learning disabilities and developed a dyskinetic movement disorder with dystonia and choreoathetosis. Her dystonia worsened and additionally she had short epileptic seizures during periods of infection. She also had visual impairment, bilateral ptosis, feeding difficulties, and vomiting. MRI of the brain was performed at four years of age and showed bilateral, symmetrical signal abnormalities in putamen and caput nucleus caudatus, a picture consistent with Leigh syndrome. A follow-up investigation at seven years of age showed progression with signal abnormalities also in mesencephalon, pons, and medulla oblongata, as well as global atrophy of the brain. Biochemical workup revealed increased concentrations of lactate in blood (max 6,5 mmol/L, ref.<2, 3) and CSF (3,6 mmol/L, ref.<2, 3). In urine, there was highly increased excretion of *α*-ketoglutaric acid and moderately increased excretion of lactic, fumaric, and glutaric acid. She died from a sudden, unexpected cardiac arrest at the age of seven years.

### 3.2. Investigations of Muscle Biopsies and Cultured Fibroblasts

**Figure 1:**
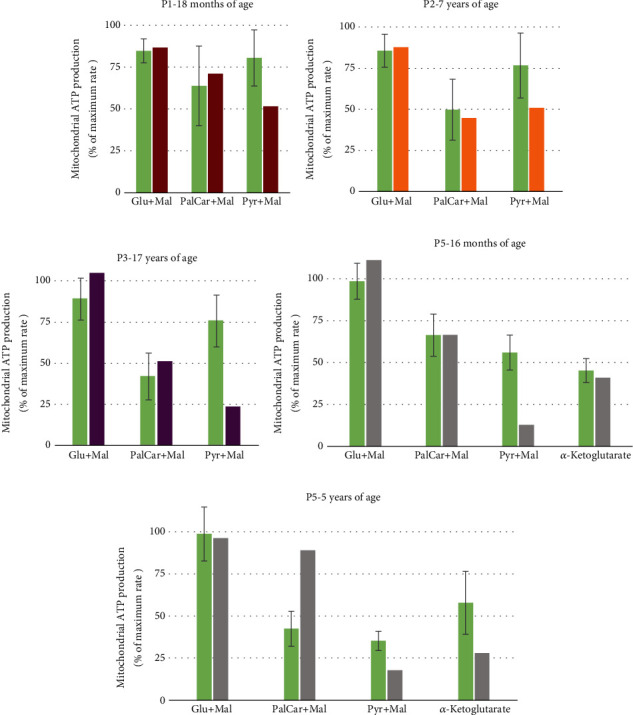
Mitochondrial ATP production (MAPR) in skeletal muscle. MAPR for patients P1, P2, P3, and P5 in the presence of glutamate+malate, palmitoyl-L-carnitine+malate, and pyruvate+malate. Patient P5 was investigated on two occasions and was the only one investigated also using the substrate *α*-ketoglutarate. Activities are expressed in percentage of maximum mitochondrial ATP production obtained in the (a–c) presence of glutamate+succinate or a (d, e) mixture of pyruvate+palmitoyl-L-carnitine+*α*-ketoglutarate+malate. The biochemical investigations were performed over several decades, under which methods and instrumentation have undergone several alterations. Consequently, each muscle biopsy is compared to an age-matched reference material (green bars) specific for the time of the investigation. Error bars indicate reference range, taken as ±2SD in each reference group.

The muscle biopsy of P1 was carried out at 1,5 years of age, P2 at 7 years of age, P3 at 17 years of age, and P5 at 16 months and 5 years of age. No muscle biopsy was conducted on P4. Mitochondrial respiratory chain (MRC) enzyme activities, as well as morphological studies of the muscle biopsy, showed normal results for all examined patients (data not presented). MAPR were decreased in all patients using the substrate combination pyruvate+malate (Figure 1), although only a mild reduction was observed in patients P1 and P2 compared to our reference values ± 2 standard deviations (Figures 1(a) and 1(b)). In the first biopsy of patient P5, MAPR was decreased for all added substrates (data not shown). The ratio between the substrates was normal despite the severely reduced activity for pyruvate+malate (Figure 1(d)). In the second biopsy performed four years later, only a reduced MAPR activity using substrate combination pyruvate+malate and using the substrate *α*-ketoglutarate was found (Figure 1(e)). This was the only patient investigated using this substrate. The activity of the total PDH complex in cultured fibroblasts from P1 and P2 showed a reduced activity, mean 0.50 and 0.45 nmol/mg protein/min, respectively, compared to controls (0.6–0.9 nmol/mg protein/min).

### 3.3. DNA Analyses

WGS analysis identified hemizygous synonymous variants in *PDHA1* in P1 and P2: c.447A>G, (p.Lys149=) in P1 and c.570C>T, p.(Cys190=) in P2. The variants were confirmed in the patients by Sanger sequencing but were absent in the parents, suggesting that they had occurred de novo. In P3, two deep intronic heterozygous variants in *PDHX* were found: c.1023+2267G>A and c.1023+2302A>G. Segregation analysis, by Sanger sequencing of samples from P3 and P4 and their parents, showed that the variants were inherited from each parent and thereby compound heterozygous in the sisters. In P5, a heterozygous missense variant, c.161A>G, p.(Asp54Gly), in *TPK1* was first identified. Extended analysis of sequence data from *TPK1* was performed and a heterozygous deep intronic variant, c.185+15054G>A, was found. Sanger sequencing of samples from the patient and parents showed that the mother carries the missense variant and the father the intronic variant, confirming that the variants were compound heterozygous in the patient. The variants c.447A>G and c.570C>T in *PDHA1* and c.161A>G and c. 185+15054G>A in *TPK1* are published earlier, but without specified phenotype of the patients or any functional studies to confirm pathogenicity of the variants [16].

### 3.4. Population Frequency and *In Silico* Analyses of the Detected Variants

The variants (c.447A>G, p.(Lys149=) and c.570C>T, p.(Cys190=) in *PDHA1*, c.1023+2302A>G in *PDHX*, and c.161A>G, p.(Asp54Gly), in *TPK1* were absent in gnomAD v4.0.0. Variant c.1023+2267G>A in *PDHX* had an allele frequency of 0.000007 (1 heterozygote) and c.185+15054G>A in *TPK1* 0.000004 (3 heterozygotes) in gnomAD v4.0.0. All variants were absent in our local database. *In silico* prediction indicated that the synonymous variants lead to loss of binding site for alternative splicing factor 2 (SRSF1) splice enhancer protein according to ESEFinder, a higher chance of exon skipping according to EX-SKIP, and an increased likelihood of intron 4 acceptor site loss for c.447A>G and intron 6 donor site loss for c.570C>T pursuant to SpliceAI. The intronic variants were predicted to lead to creation of either a new donor site or acceptor site as per all prediction tools in Alamut as well in SpliceAI. The binding site sequences for ESEs and scores from the splicing prediction tools for the synonymous variants in *PDHA1* are presented in Table 1. The sequences and prediction scores of the splice sites created by the variants and activated splice sites in *PDHX* and *TPK1* are shown in Table 2. Missense variant c.161A>G (p.(Asp54Gly) in *TPK1* was predicted as possibly damaging by Polyphen (HumDiv score = 0.909 and HumVar score = 0.521), predicted tolerated by SIFT (score = 0.26), and had a CADD score of 22.

### 3.5. RNA Analyses

#### 3.5.1. *PDHA1* RNA Analysis

**Figure 2:**
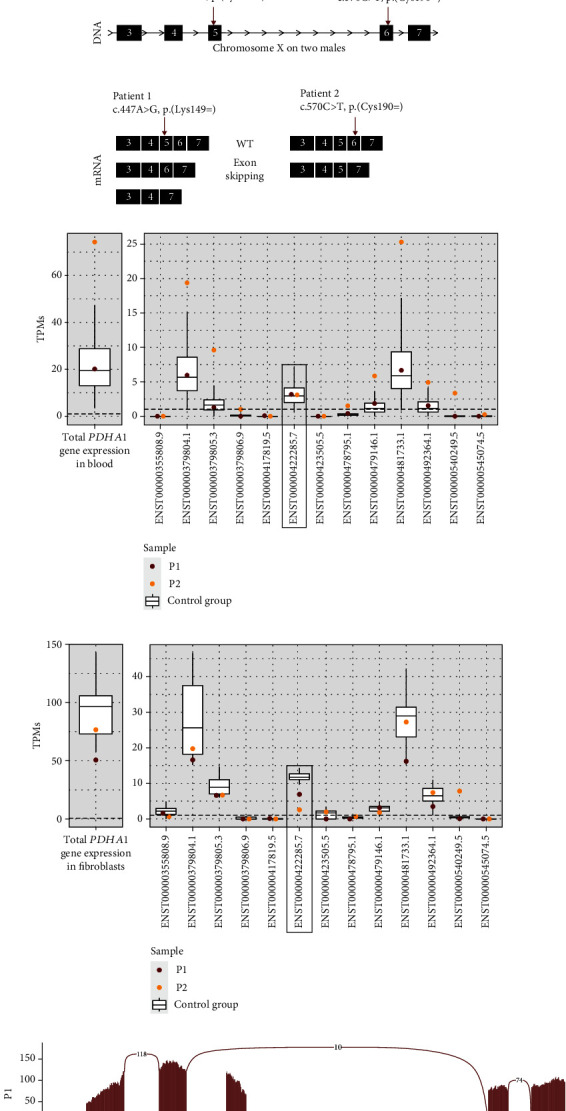
RNA analyses of *PDHA1* in RNA from blood and cultured fibroblasts. (a) PCR products of *PDHA1* cDNA generated from RNA extracted from patient 1 (P1) and control (C) fibroblasts, treated with (+) and without (-) emetine 7 hours before harvesting, and RNA extracted from blood from P1 and C. Extra bands in P1 correspond to mRNA lacking exon 5 and exon 5+6. (b) PCR products of *PDHA1* cDNA generated from RNA extracted from blood from patient 2 (P2), his mother, father, and control (C) and RNA extracted from fibroblasts from P2 and control, treated with (+) and without (-) emetine 7 hours before harvesting. Extra band in P2 corresponds to mRNA lacking exon 6. (c) Schematic picture of variants identified in P1 and P2 *PDHA1* on DNA level and the resulting aberrant spliced mRNAs. (d) Gene expression of *PDHA1* in blood and total expression/all different known transcripts separately of 135 healthy controls, P1, and P2; framed box plot indicates MANE Select transcript (NM_000284.4, ENST00000422285.7); transcripts with high expression in P2 are naturally occurring transcripts without exon 6: ENST00000379804.1 (exons 8-9-10-11), ENST00000379805.3 (exons 1-2-3-4-5), ENST00000481733.1 (exons 7-8-9-10), ENST00000492364.1 (exons 1-2-3-4), and ENST00000540249.5 (exons 1-2-3-4-5-7-8-9-10-11). (e) Gene expression of *PDHA1* in fibroblasts and total expression/all different known transcripts separately of 21 healthy controls, P1, and P2; framed box plot indicates MANE Select transcript (NM_000284.4, ENST00000422285.7); transcript with high expression in P2 is naturally occurring transcript without exon 6: ENST00000540249.5 (exons 1-2-3-4-5-7-8-9-10-11). (f) Sashimi plot of RNAseq data from fibroblast RNA from P1, P2, and the control group consisting of the median number of reads in 3 healthy controls. RNA coverage is given on the *y* axis, and a number of split reads spanning introns are indicated on the exon junction lines. Bottom graph depicts MANE Select transcript (NM_000284.4, ENST00000422285.7) and known transcript (NM_001173456.2, ENST00000540249.5) lacking exon 6 with reference exons as black boxes. Plot shows a low proportion of aberrantly spliced mRNA lacking exon 5 in P1 and high proportion of aberrantly spliced mRNA lacking exon 6 in P2. The control group has a small number of splice junctions directly from exons 5 to 7, corresponding to the known transcript lacking exon 6.

Analysis of *PDHA1* cDNA from RNA isolated from blood from P1 showed alternative transcripts in the patient in comparison to control (Figure 2(a)). The shorter weak fragments in the patient corresponded to cDNA lacking exon 5 and exons 5-6. In RNA from cultured fibroblasts, however, only wild-type product was detected. On the other hand, RNA isolated from cultured fibroblasts treated with emetine showed a high proportion of the product corresponding to the truncated cDNA, missing exons 5-6, and a lower proportion of the product corresponding to wild type, indicating that the aberrant spliced transcripts in the nontreated cells was subject to nonsense-mediated decay (NMD).

In P2, *PDHA1* cDNA analysis on RNA isolated from blood showed a shorter fragment, corresponding to a fragment lacking exon 6, in addition to the expected product observed in parents of P2 and a control sample (Figure 2(b)). Analysis of RNA isolated from cultured fibroblasts, with or without treatment of emetine, showed the same result, and the proportion of the product corresponding to the truncated cDNA missing exon 6 was high and the proportion of the product corresponding to wild type was low under both culture conditions (Figure 2(b)). The variants of P1 and P2 on DNA level and the resulting products on mRNA level are schematically pictured (Figure 2(c)).

RNAseq analysis of blood mRNA from P1 detected normal expression levels of total *PDHA1* and normal expression of all individual isoforms (Figure 2(d)). In contrast, P2 presented with normal expression levels of the MANE Select transcript (NM_000284.4, ENST00000422285.7) but increased steady-state levels of other known isoforms. Interestingly, these latter isoforms naturally lack exon 6, resulting in an overall increase of *PDHA1* expression (Figure 2(d)). Normal expression of the MANE Select transcript in blood in both P1 and P2 corresponds well with the weak splice defect seen in blood in the cDNA analysis. However, expression of the MANE Select transcript is below the normal range in cultured fibroblasts from both P1 and P2 (Figure 2(e)). In similarity with the blood analysis, the expression of transcripts lacking exon 6 is higher in P2 than P1 fibroblasts, which also gives rise to an expression in the normal range for P2 when looking at total gene expression (Figure 2(e)).

Sashimi plots of RNAseq data from fibroblasts from P1, P2, and a control group showed 353 splice junction reads from exons 5 to 7 and only 104 splice junction reads from exons 5-6 in P2, confirming the results of the cDNA analysis with high proportion of transcripts lacking exon 6 (Figure 2(f)). In P1, there is an almost normal splicing with only 10 splice junction reads from exons 4 to 6 and 110 splice junction reads from exons 4 to 5 and 62 from exons 5-6 (Figure 2(f)), like the result of the cDNA analysis. For P1, only the cDNA analysis on RNA from emetine-treated cells captured the aberrant splicing (Figure 2(a)).

#### 3.5.2. *PDHX* RNA Analysis

**Figure 3:**
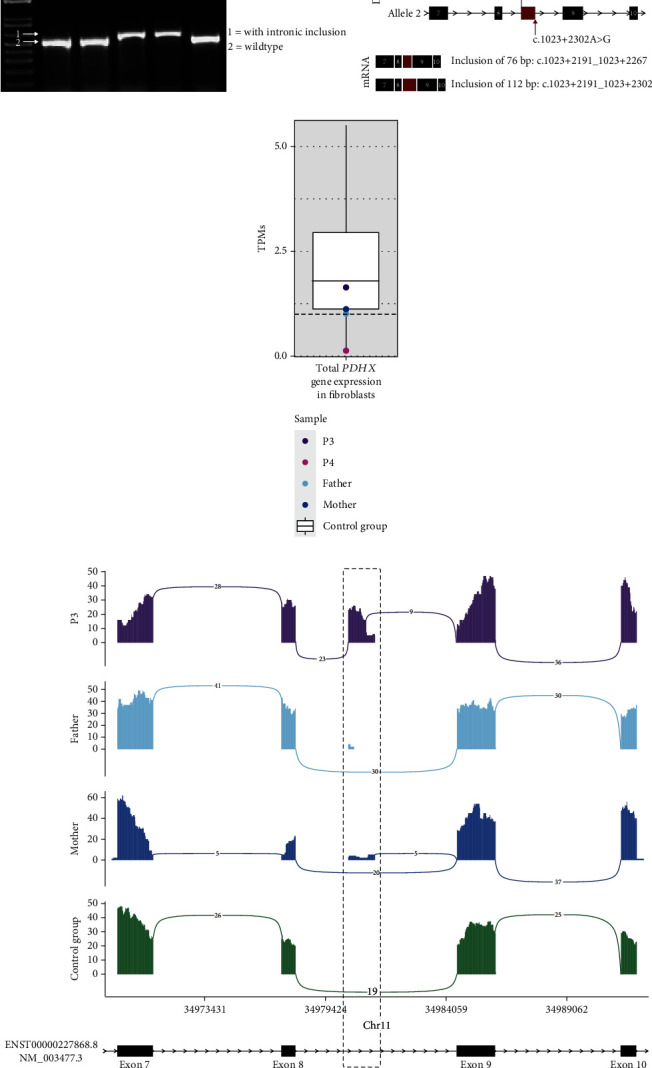
RNA analyses of *PDHX* in RNA from blood. (a) PCR products of *PDHX* cDNA generated from RNA from patient 3 (P3), her affected sister (P4), parents, and control (C) blood, showing longer products than wild type in the patients. (b) Schematic picture of variants identified in P3 and P4 *PDHX* on DNA level and the resulting aberrantly spliced mRNAs. (c) Box plot showing total gene expression of *PDHX* in blood in P3 and P4 and their parents compared to the control group of 135 healthy individuals. (d) Sashimi plot of RNAseq data from blood from P3, her parents, and the control group consisting of the median number of reads in 10 healthy controls. RNA coverage is given on the *y* axis, and a number of split reads spanning introns are indicated on the exon junction lines. Bottom graph depicts MANE Select transcript (ENST00000227868.8, NM_003477.3) with reference exons 7-10 as black boxes. The dashed box marks the position of the included pseudoexon from intron 9. No wild-type splice junctions from exons 8 to 9 are visible in P3.

Analysis of *PDHX* cDNA extracted from blood from P3, her affected sister P4, their parents, and a control showed a slightly longer product in both patients (Figure 3(a)). The longer product corresponds to cDNA with inclusion of intronic sequence. The variants c.1023+2267G>A and c.1023+2302A>G both create new donor sites as predicted by the *in silico* analyses. These novel donor sites in turn both activate an acceptor site at position c.1023+2191, as predicted by the *in silico* tools (Table 2). This results in pseudoexon inclusion of 76 bp and 112 bp on each mRNA allele (Figure 3(b)).

Sashimi plots of RNAseq data from RNA isolated from blood from P3, the parents and a control group, show inclusion of a pseudoexon in intron 8 at the expected position from the result of the cDNA analysis. In the plot of P3 no normal exon 8 to 9 junctions are seen, only aberrant splicing from exon 8 (23 junctions) and exon 9 (9 junctions) to the pseudo-exon are visible (Figure 3(d)). RNAseq expression analysis shows a total gene expression of *PDHX* in the normal range for P3 and a very low expression for P4 (Figure 3(c)).

#### 3.5.3. *TPK1* RNA Analysis

**Figure 4:**
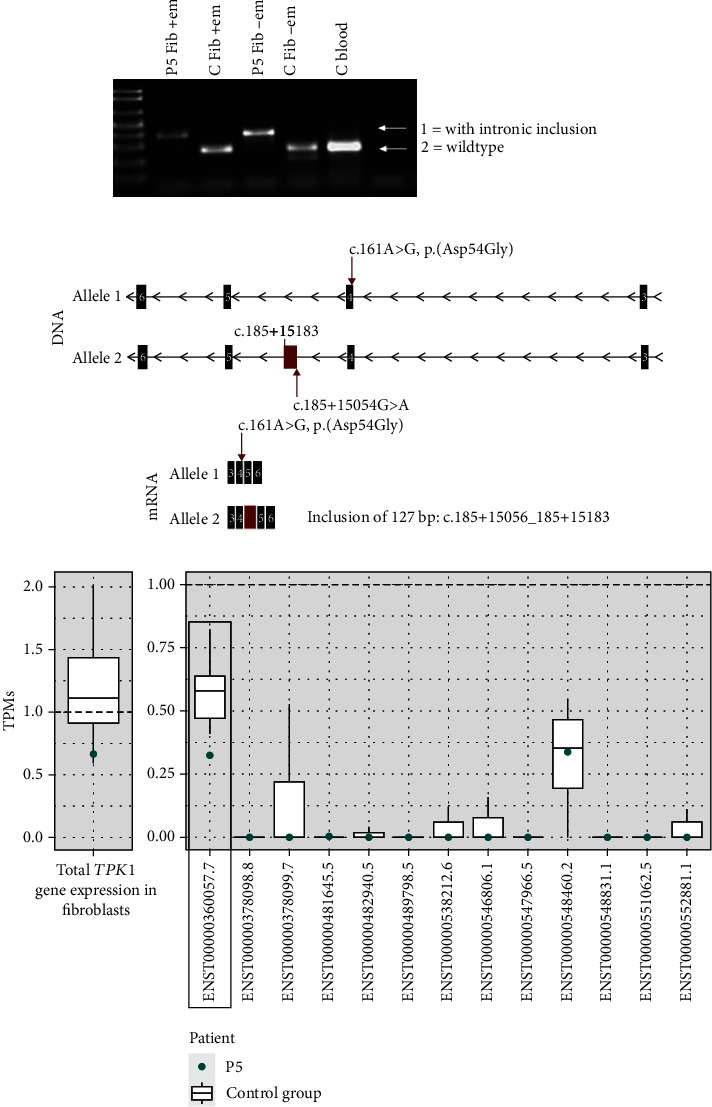
RNA analyses of *TPK1* in RNA from cultured fibroblasts. (a) Allele-specific (allele of father with c.185+15054G>A) PCR products of *TPK1* cDNA generated from RNA extracted from patient 5 (P5) and control (C) fibroblasts, with (+) and without (-) emetine treatment 7 hours before harvesting, and C blood, showing longer products in the patient's samples. (b) Schematic picture of variants identified in P5 *TPK1* on DNA level and the resulting aberrantly spliced mRNAs. (c) Box plot showing gene expression of *TPK1* in blood and total expression/all different known transcripts separately of P5 and 22 healthy controls. Framed box plot indicates MANE Select transcript (NM_022445.3, ENST00000360057.7).

For *TPK1* cDNA analysis, parental RNA samples were unfortunately not available. Amplification was only performed on cDNA derived from cultured fibroblasts (treated with/without emetine) from the patient and a control. Analysis of cDNA (exons 1 to 7) did not give conclusive results (not shown). Thus, allele-specific primers were designed and used for further analysis. Amplification of the allele of the father (containing the deep intronic variant c.185+15054G>A) showed a longer product in the patient in comparison with control in samples from both emetine-treated and nontreated cells (Figure 4(a)). The longer product corresponds to cDNA with inclusion of 127 bp intronic sequence. The variant c.185+15054G>A creates a novel splice acceptor site, which leads to an existing donor site at position c.185+15183 being activated, as predicted by the *in silico* analysis (Table 2), leading to inclusion of a pseudoexon between the two splice sites (Figure 4(b)). Amplification of the allele of the mother showed products with expected length in the patient samples but no products in the control as expected (not shown).

RNAseq analysis of *TPK1* from RNA isolated from fibroblasts from P5 shows a low expression of the MANE Select transcript (NM_022445.3, NM_ENST00000360057.7) in the patient (Figure 4(c)) compared to the control group. It is not possible to show the aberrant splicing in a sashimi plot from RNAseq data for this case since the overall expression of *TPK1* in fibroblasts is too low for the analysis. For this gene, it would have been better to perform the analysis in RNA from blood, as the expression is higher there, but this was not possible since the patient is deceased.

## 4. Discussion

Diagnostics of genetic diseases have been dramatically improved thanks to next-generation sequencing (NGS). In our study at Genomic Medicine Center Karolinska-Rare Diseases (GMCK-RD) [16], clinical WGS provided a molecular diagnosis to more than 1200 patients with rare diseases during the years 2015-2019. Overall, 40% of the patients included in the study got a molecular diagnosis. For the group of patients with metabolic diseases (including mitochondrial diseases), 32% received a genetic diagnosis in a previously identified known disease gene. WGS, however, can also detect variants in control regions as well as deep intronic and synonymous variants. For these and other variants of uncertain significance (VUS), functional studies are required for evaluation of pathogenicity.

In the present study, we demonstrate that novel synonymous variants in the gene *PDHA1* lead to partial exon skipping with a subset of the transcripts remaining correctly spliced. In addition, we confirm that deep intronic variants in the genes *PDHX* and *TPK1* lead to pseudoexon inclusion in mRNA. The variants in *PDHA1* identified in P1 and P2 are synonymous, but the fact that they both were (1) shown to be de novo, (2) predicted to change ESE motifs in exons 5 and 6, (3) not found in the normal population, and (4) ATP production was decreased, with substrates malate+pyruvate in mitochondria isolated from the patient's muscle, suggested that the variants were causing PDCD in the patients. Analysis of the patients' cDNA from blood indicated partial exon skipping caused by the variants. Since pre-mRNAs with skipped exons are most probably targeted for nonsense-mediated decay (NMD), to prevent formation of truncated proteins, complementary analyses of cDNA from cultured fibroblasts, treated with and without emetine were performed. Emetine is a translation inhibitor which can be used to block NMD in cell lines [29]. For variant c.447A>G treatment with emetine was essential to visualize the defect. The splice-defect was not detectable at all in the nontreated fibroblasts, but in the analysis of emetine-treated cells, there were clear bands corresponding to truncated mRNA. This suggests that the reason for not detecting the mis-spliced mRNAs is because they are subjected to NMD, which also is supported by the overall low detected expression of *PDHA1* in the fibroblasts from this patient. For the variant c.570A>T, emetine treatment did not make any difference, but the splice defect was much more pronounced in fibroblasts than in blood, with a smaller proportion corresponding to wild type in fibroblasts regardless of treatment. RNAseq confirmed the result of low expression of MANE Select transcript and a high expression of transcripts lacking exon 6. The reason for the difference in degradation of mRNA in the two patients is most probably because exon 5 skipping cause a shift in reading frame and introduction of a premature termination codon, leading to NMD of this mRNA, compared to exon 6 skipping where the reading frame is preserved, causing this mis-spliced mRNA to escape NMD. The observed increased levels of *PDHA1* transcripts lacking exon 6 in P2 could be caused by a compensatory response due to either increased expression of the gene or decreased degradation or a combination of both. PDH activity in cultured fibroblasts from P1 and P2 showed a decreased activity, around 60% of normal activity, higher than typically expected for males with pathogenic variants in *PDHA1*, where residual activity for most cases earlier has been found to be between 20% and 50% [32].

This result is consistent with the observation that there are both correctly and incorrectly spliced *PDHA1* transcripts and that there is a variation between different tissues. Most likely, this can also explain why the two boys have a milder clinical picture than males with pathogenic variants in *PDHA1* usually have.

More than 250 disease-causing variants in *PDHA1* have been described to date [33] (Professional HGMD database, accession date August 8, 2023). Most of them are missense variants or deletions/insertions spread within all exons. Only a handful are variants located within splice junction canonical sequences. Several are, however, exonic synonymous [34–36], missense [35, 37, 38], and nonsense [39] variants in exon 5, 6, or 7 shown to cause aberrant splicing of these exons or different combinations of them. All these variants are predicted to disrupt different ESE motifs in the exons. ESEs are of particular importance in genes with weak splice sites and variants in these elements often lead to exon skipping of the exon in which they are positioned, and the proportion of aberrant splicing may be connected to the strength of the surrounding splice sites. The acceptor and donor sites of intron 5 have the lowest scores, 72 and 75 by SSF prediction tool, compared to all other introns in the *PDHA1* gene, most of which have scores above 80. The prediction scores for the splice sites surrounding exons 5 and 6 are presented in Table 3. This probably explains the aberrant splicing of exon 6 caused by a nonsense variant (c.729C>A) occurring in exon 7 and synonymous variants (c.523G>A and c.555A>G) in exon 6 [39] and in our study, aberrant splicing of exon 5 and/or 6 caused by synonymous variants in exons 5 and 6 as these variants are predicted to further weaken correct splicing. SpliceAI predicts a weakening of the acceptor site of intron 4 by the variant in exon 5 and a weakening of the donor site of intron 6 by the variant in exon 6 (Table 1), which most probably together with the existing weak acceptor and donor sites in intron 5 lead to exon 5 and/or exon 6 skipping (Table 3). *In vitro* studies by Ridout et al. [39] have shown that artificial strengthening of the intron 5 acceptor site in a sequence where c.729C>A is present leads to normal splicing of exon 6. The same results were obtained in sequences with variants c.523G>A and c.555A>G, which completely abolished exon 6 skipping with an artificially strengthened acceptor site [39]. They suggest that ESEs may affect splicing not only of the exon in which they occur but also neighboring exons, which is also supported by our results.

The clinical picture of the patients P3 and P4 and the clearly decreased MAPR, with substrates malate+pyruvate in mitochondria isolated from muscle from P3, suggested a diagnosis of PDCD in the sisters already in the year 2003, but the deep intronic variants in *PDHX* were identified by WGS in 2020. Both variants are shown to lead to intronic inclusions. Total gene expression of *PDHX* was very low in P4 but still in the normal range for P3, indicating that the aberrant spliced mRNA in blood in P4 at a higher grade is subject for NMD than in P3. The reason for this is unclear. Activation of novel donor or acceptor sites is a common known disease mechanism of deep intronic variants next to changing of ISE and ISS motifs in the intronic sequence [10]. The identified variants are the first deep intronic variants described in *PDHX*. So far, only 23 variants are reported in this gene; two are missense variants and the rest are nonsense, canonical splice site variants, and deletions/insertions within exons [33] (Professional HGMD database, accession date August 8, 2023).

The clinical picture and low MAPR, with substrates malate+pyruvate and *α*-ketoglutarate in mitochondria isolated from muscle, raised the suspicion of a mitochondrial disease in P5 early during the medical investigation, but the girl unfortunately did not get a genetic diagnosis until year 2020, 23 years after her death. The WGS analysis at first identified a heterozygous missense variant in *TPK1*. This finding prompted a further analysis of the gene to search for a possible second disease-causing variant in the intronic parts of the gene, since TPK deficiency would be an appropriate explanation for the disease. It would explain low MAPR with both the substrates malate+pyruvate and *α*-ketoglutarate and high levels of *α*-ketoglutarate in the urine, since TPK is the enzyme which catalyzes the conversion of thiamine (vitamin B1) to thiamine pyrophosphate (TPP). TPP is a cofactor for both pyruvate dehydrogenase and *α*-ketoglutarate dehydrogenase. The analysis detected a deep intronic variant which was shown to lead to inclusion of intronic sequence in the mRNA. The variant is the first reported deep intronic variant in *TPK1*, only 26 variants are reported in this gene, and most of them are missense variants [33] (Professional HGMD database, accession date August 8, 2023).

## 5. Conclusions

In summary, we identified synonymous and deep intronic variants causing primary and secondary PDCD. These results provide new insight, expand the diversity of genetic variants causing PDCD, and highlight the importance of atypical variants leading to defective splicing. *In silico* predictions and RNA analysis, both cDNA analysis and RNAseq, are important and necessary for determining the effects of these types of variants. The severity of the splice defects and resulting biochemical dysfunction varied between tissues, stressing the importance of performing biochemical and transcript analysis in affected tissues. Moreover, the splice defect caused by one of the variants in *PDHA1* could only be detected when NMD was blocked by treatment of the cells with emetine. Importantly, the result of the RNAseq expression analysis is misleading when looking at total gene expression, which for P2 showed a very high expression. However, when examining the MANE Select transcript separately, it is much lower than the normal range of expression. The two males with hemizygous synonymous *PDHA1* variants also have a mild phenotype and higher PDH enzyme activity than expected, which is consistent with aberrant but leaky splicing with a proportion of the transcripts remaining correctly spliced.

## Figures and Tables

**Table 1:** Binding site sequences for ESEs and *in silico* prediction scores of synonymous variants in *PDHA1*.

Splicing prediction tool	*PDHA1* c.447A>G, p.(Lys149=)		*PDHA1* c.570C>T, p.(Cys190=)	
	WT	Variant	WT	Variant
ESEFinder SRSF1 (SF2/ASF)	2,09 (GAAAGGA)	0	2,21 (CTGCCTG)	0
RESCUE_ESE	1 (AAAGGA)	0	0	0
EX-SKIP (ESS/ESE ratio)	0,22	0,33	0,54	0,67
SpliceAI	NA	*Δ*0,49 (intron 4 AL)	NA	*Δ*0,33 (intron 6 DL)

Abbreviations: AL = acceptor site loss; DL = donor site loss; ESE = exonic splicing enhancer; ESS = exonic splicing silencer; NA = not applicable; SRSF1 (SF2/ASF) = alternative splicing factor 2; WT = wild type. The nucleotides changed by the variants are underlined. Reference sequence: RefSeq NM_000284.4.

**Table 2:** Sequence and *in silico* prediction scores for splice sites created by the variants and the activated splice sites.

Position		Sequence	SSF (0-100)	MaxEnt (0-16)	NNSPLICE (0-1)	GeneSplicer (0-21)	SpliceAI (0-1)
*PDHX* c.1023+2267A>G	WT	AAG/ATAATAGATT	0	0	0	0	NA
	Variant (DS)	AAG/GTAATAGATT	81,97	8,49	0,93	3,72	*Δ*0,99
*PDHX* c.1023+2302A>G	WT	CCC/ATAAGTCTTC	0	0	0	0	NA
	Variant (DS)	CCC/GTAAGTCTTC	77,77	9,09	0,98	0	*Δ*0,94
*PDHX* c.1023+2191	Activated AS	TTTTGAGCAG/CAG	0	5,91	0	0,8	NA
*TPK1* c.185+15054G>A	WT	TCTTTCTTGG/GTA	0	0	0	0	NA
	Variant (AS)	TCTTTCTTAG/GTA	88,46	12,03	1,0	12,65	*Δ*0,99
*TPK1* c.185+15183	Activated DS	GAG/GTGAAGACCA	75,41	4,41	0	1,38	NA

Abbreviations: AS = acceptor site; DS = donor site; NA = not applicable; SSF = SpliceSiteFinder-like; WT = wild type. The nucleotides changed by the variants are underlined. Reference sequences: RefSeq NM_003477.3 for *PDHX* and NM_022445.3 for *TPK1.*

**Table 3:** Sequence and *in silico* prediction scores for the splice sites surrounding exon 5 and exon 6 in *PDHA1*.

Splice site	Sequence	SSF (0-100)	MaxEnt (0-16)	NNSPLICE (0-1)	GeneSplicer (0-21)
Intron 4 AS	CCATTTCCAG/GAC	89,51	9,81	0,86	9,65
Intron 5 DS	CAG/GTAGTCAAGG	71,75	5,30	0,70	2,40
Intron 5 AS	TTAATGTTAG/GTG	74,81	6,92	0,93	3,33
Intron 6 DS	CAG/GTAATTATGT	87,61	8,55	0,99	1,72

Abbreviations: AS = acceptor site; DS = donor site; SSF = SpliceSiteFinder-like; WT = wild type.

## Data Availability

The data that support the findings of this study is available upon reasonable request from the corresponding author.
